# All around the Year

**Published:** 1892-02

**Authors:** 


ALL AROUND THE YEAR. A Calendar for 1892. Boston: Lee & Shepard, 1892. Price, 50 cents.

This calendar consists of a series of cards—one for each month—containing the calendars for the months, and colored illustrations of an infant in various amusing attitudes. Very pretty as a souvenir and Christmas card.

